# *Bergeyella cardium*: Clinical Characteristics and Draft Genome of an Emerging Pathogen in Native and Prosthetic Valve Endocarditis

**DOI:** 10.1093/ofid/ofz134

**Published:** 2019-03-15

**Authors:** Jennifer S Mulliken, Charles Langelier, Jehan Z Budak, Steve Miller, David Dynerman, Samantha Hao, Lucy M Li, Emily Crawford, Amy Lyden, Michael H Woodworth, Joseph L DeRisi, Edward Desmond, Christina Browne, Araceli Luu, Donald J Grandis, William Grossman, Tobias Deuse, Gregory P Melcher

**Affiliations:** 1 Division of Infectious Diseases, University of California, San Francisco; 8 Division of Cardiology, University of California, San Francisco; 9 Division of Adult Cardiothoracic Surgery, University of California, San Francisco; 2 Department of Laboratory Medicine, University of California, San Francisco; 4 Department of Microbiology and Immunology, University of California, San Francisco; 6 Biochemistry and Biophysics, University of California, San Francisco; 3 Chan Zuckerberg Biohub, San Francisco; 7 California Department of Public Health, Microbial Diseases Laboratory, Richmond; 5 Division of Infectious Diseases, Emory University, Atlanta, Georgia

## Abstract

*Bergeyella cardium* is a new species in the family Flavobacteriaceae that was recently described in 3 cases of native valve infective endocarditis. We report the first case of *B. cardium* prosthetic valve endocarditis, provide the first draft genome of this species, and review the microbiologic characteristics of this emerging pathogen.

## CASE REPORT

Advanced molecular diagnostics are increasingly being used to aid in the diagnosis and treatment of infectious diseases. Here we present a case of prosthetic valve infective endocarditis caused by *Bergeyella cardium* and provide both antimicrobial susceptibility testing and whole genome sequencing phylogenetic analysis for this unusual emerging pathogen. An 81-year-old man with a history of bioprosthetic aortic valve replacement 6 years earlier was admitted with several months of fever, night sweats, and malaise. He had been in his usual state of good health until 3 months before admission, when he began experiencing intermittent chills, night sweats, and exertional shortness of breath. He denied recent travel and reported no history of animal contact, occupational exposures, or intravenous drug use. Aside from a routine dental cleaning 3 months earlier, before which he took amoxicillin, he denied any recent dental procedures. 

Blood cultures obtained when he was an outpatient were negative for growth on bacterial and mycobacterial media (BacT/Alert; bioMérieux). His symptoms subsided after a course of azithromycin, but in subsequent weeks the patient noted worsening fatigue followed by progressive exertional dyspnea. Another set of blood cultures obtained 1 week before hospital admission yielded no growth. His fever recurred, and he was admitted to our quaternary care hospital after a transthoracic echocardiogram revealed thickened aortic valve leaflets concerning for prosthetic valve endocarditis.

On arrival at the hospital, the patient had a fever of 38.5°C. Examination revealed a systolic murmur at his right upper sternal heart border with radiation to the clavicle. His lungs were clear to auscultation, and he had no peripheral stigmata of endocarditis or signs of heart failure. Laboratory studies were notable for a creatinine level of 1.37 mg/dL (reference range, 0.61–1.24 mg/dL) and a C-reactive protein level of 88 mg/L (reference range, <6.3 mg/L). Transesophageal echocardiography showed a mean prosthetic valve gradient of 51 mm Hg, an aortic valve area of 0.63 cm^2^, and severe prosthetic valve leaflet restriction concerning for vegetation or thrombus. No abscess or vegetation was seen, however, and the prosthetic valve showed no signs of dehiscence. 

Empiric ceftriaxone treatment was started, and the patient was hospitalized for 3 days. Eight sets of routine blood cultures analyzed using the BACTEC system and 2 sets of mycobacterial blood cultures obtained before antibiotic treatment remained negative for growth. Given the lack of growth in multiple sets of blood cultures, endocarditis due to staphylococcal, streptococcal, and HACEK pathogens (*Haemophilus spp, Aggregatibacter spp, Cardiobacteriugm hominis, Eikenella corrodens, and Kingella spp*) was considered unlikely. Although the patient denied any animal exposures, the chronicity of his illness, 10 negative sets of blood cultures over several weeks’ time, and the waxing and waning nature of his symptoms raised concern for Q fever endocarditis. As a result, he was treated empirically for *Coxiella burnetii* with doxycycline and hydroxychloroquine while awaiting serologic results.

Three days after the patient was discharged to home, 5 blood cultures from his hospitalization turned positive. Gram stain revealed gram-negative coccobacilli. The organism grew poorly on solid media and was not identified by means of colony mass spectroscopy (matrix-assisted laser desorption ionization time-of-flight mass spectrometry; Bruker) because the species was not in the database. The patient’s constitutional symptoms initially resolved with antibiotic treatment, and repeated blood cultures were sterile. Two weeks after discharge, however, he began experiencing worsening fatigue and dyspnea on exertion, concerning for acute symptomatic heart failure. An electrocardiogram revealed new first-degree atrioventricular and left bundle branch blocks. Transthoracic echocardiography showed new severe left atrial enlargement as well as a mean aortic valve gradient of 96 mm Hg. 

The patient was subsequently readmitted to the hospital to receive a bovine aortic valve replacement. Based on blood culture results, therapy was changed to ceftriaxone at 2 g/d. Intraoperatively, thrombotic pannus covering the bioprosthetic aortic valve leaflets was noted. No abscess was seen. The pannus was removed and sent for culture as well as polymerase chain reaction testing using primers for bacterial 16S ribosomal DNA (rDNA) gene detection. Postoperative blood cultures were sterile, and valvular tissue showed no growth on bacterial, fungal, and mycobacterial cultures. Results of serologic testing for *Bartonella* spp., *Brucella* spp., and *C. burnetii* were ultimately negative.

Three weeks after surgery, the gram-negative coccobacillus observed on blood cultures was identified as *Bergeyella cardium* by the California Department of Public Health, using 16S rDNA gene sequencing. Results of bacterial 16S rDNA gene polymerase chain reaction testing on the aortic valve tissue were also positive for *B. cardium*. After surgery, the patient completed a 6-week course of ceftriaxone, with symptom resolution and normalization of serum inflammatory marker levels. Blood cultures obtained 2 weeks after completion of therapy remained negative.

## ISOLATE CHARACTERISTICS

Similar to previously reported *B. cardium* strains, this isolate demonstrated slow growth on blood and chocolate agar and was negative for indole and oxidase production. Susceptibility testing of the bacterial isolate on Mueller-Hinton blood agar using the Etest method showed susceptibility to penicillins, cephalosporins, fluoroquinolones, carbapenems, and tetracyclines. Although there are no Clinical and Laboratory Standards Institute interpretation criteria for *B. cardium*, these values are in the susceptible range for HACEK group organisms using the 2015 Clinical and Laboratory Standards Institute interpretive criteria [1]. Additional antimicrobial susceptibilities are included in Table 1.

**Table 1.** Antimicrobial Susceptibilities of *Bergeyella cardium* Isolate Tested Using Kirby-Bauer and Etest Methods

Antibiotic	Kirby-Bauer Susceptibility, mm	Etest Susceptibility, μg/mL
Amikacin	12	…
Aztreonam	28	2
Cefepime	42	…
Ceftazidime	39	…
Ceftazidime-avibactam	…	0.064
Ciprofloxacin	27	0.5
Doripenem	…	0.064
Ertapenem	…	0.064
Levofloxacin	26	0.5
Meropenem	47	0.094
Minocycline	…	0.064
Piperacillin-tazobactam	50	…
Tetracycline	…	0.5
Tobramycin	6	…

**Figure 1.**
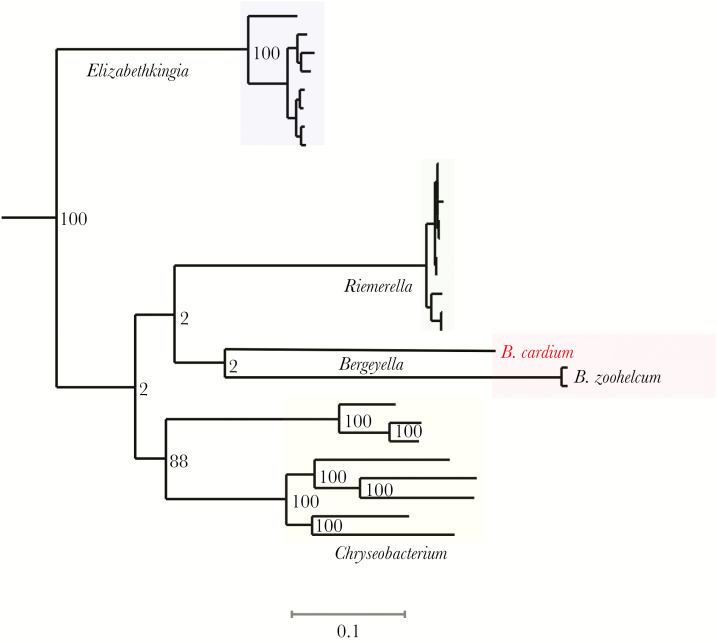
Phylogenetic tree created using the PATRIC database [2], based on alignment of protein sequences shared across all genomes. Numbers on the branches represent how many times the same cluster was observed when 100 trees were analyzed with 50% of the original genes. Scale bar represents 0.1 substitution per site.

Because no reference genomes were available for this species, we performed both short-read (Illumina) and long-read (Oxford Nanopore) whole-genome sequencing followed by de novo assembly to generate a 1.96-Mb draft genome consisting of 3 contigs (GenBank accession no. QYCZ00000000.1; Supplementary Material). Genome annotation (Supplementary Material) and phylogenetic evaluation demonstrated closest relatedness to *Bergeyella zoohelcum*, *Riemerella anatipestifer,* and *Elizabethkingia meningoseptica* (Figure 1) [2]. Compared with *B. zoohelcum* American Type Culture Collection 43767, *B. cardium* had an average nucleotide identity of 76.9% across the genome, a 98.1% identity across the 16S ribosomal RNA gene, and a higher GC content (39.8% vs 36.1%). Metabolic pathway analysis (Supplementary Methods) indicated fewer xenobiotic metabolism genes compared with *B. zoohelcum* and did not identify genes related to steroid, phenylpropanoid, or bile acid biosynthesis but did predict capacity for N-glycan biosynthesis, in contrast to *B. zoohelcum.* Antimicrobial resistance gene assessment performed using short-read sequence type 2 did not return any alignments, consistent with phenotypic pansusceptibility [3].

## Discussion

The family Flavobacteriaceae is a large and diverse group of gram-negative, non–spore-forming bacilli that relies primarily on aerobic metabolism. It comprises hundreds of species found in a variety of natural habitats, often in association with animals or plants. Most species are nonpathogenic in humans; however, organisms such as *Capnocytophaga canimorsus* and *E. meningoseptica* are known to cause disease, especially in immunocompromised hosts. Recent reports of other human pathogenic bacteria in the family highlight our evolving understanding of this heterogeneous group of organisms. *B. cardium* is a recently described pathogenic species of Flavobacteriaceae that has been associated with 2 reported cases of native valve infective endocarditis in Korea [4] and 1 in China [5]. To date, no cases involving this bacterium have been reported outside Asia, and to our knowledge, this is the first case of *B. cardium* prosthetic valve endocarditis described in the literature. Here we report previously undescribed microbiologic characteristics of *B. cardium* as well as a draft genome to advance understanding of this pathogen.

When initially described in 1994, the genus *Bergeyella* was distinguished from the closely related genus *Weeksella* by its urease activity, growth properties, and predominance of branched-chain fatty acids. *B. zoohelcum*, the only species in the genus at that time, was known to colonize the upper respiratory tract of dogs, but its pathogenicity and clinical significance were not well understood. Since then, there have been several case reports of *B. zoohelcum* infections published in the literature, mainly in the setting of animal bites or animal exposures. These include reports of cellulitis [6], skin abscess [7], bacteremia after a dog bite [8], and bacteremia after consumption of food made with goat blood [9]. The most recent of these *B. zoohelcum* cases occurred in a young Chinese woman with bacteremia and infective endocarditis; the latter had not previously been reported in association with *B. zoohelcum* infection [10].

When *B. cardium* was first reported in 2015 as the causative pathogen in 2 cases of native valve endocarditis, it was identified as a distinct species from *B. zoohelcum* on the basis of partial 16S ribosomal RNA gene sequencing [4]. Using whole-genome sequencing, we have built on these prior reports by more comprehensively evaluating the phylogenetic relatedness of *B. cardium* to other members of the family Flavobacteriaceae and by constructing the first draft genome of this emerging pathogen.

In conclusion, with the more widespread use of advanced molecular diagnostics to identify fastidious bacterial species, an increasing number of pathogens have been implicated in both native and prosthetic valve endocarditis. *B. cardium* is a recent example, and it is likely that more cases exist than have been reported. Although most human *Bergeyella* infections have occurred in the setting of an animal exposure, cases of *Bergeyella* endocarditis, including the case reported here, do not have clear zoonotic transmission. Our patient did, however, undergo routine dental cleaning in the months before the development of his symptoms, which raises the possibility that *B. cardium* may be part of the human oral microbiome, as *B. zoohelcum* is for dogs and cats [6–8].

Virulence determinants of *Bergeyella* species are incompletely understood and are an emerging area of research. Recently, *pncA*, which encodes a nicotinamidase in the related species *R. anatipestifer,* was identified as an important virulence factor and determinant of invasive cardiac disease in animal models [11]. We identified a related nicotinamidase gene with 71% nucleotide similarity; it is possible that this gene also serves as a key virulence determinant in *B. cardium*. This and many additional questions about this new species remain outstanding, and the incidence of *B. cardium* infections is unknown. This report not only helps clarify the role of *B. cardium* in native and prosthetic valve infections, but it also provides the first draft genome of this unusual and fastidious emerging endocarditis pathogen.

## Supplementary Data

Supplementary materials are available at *Open Forum Infectious Diseases* online. Consisting of data provided by the authors to benefit the reader, the posted materials are not copyedited and are the sole responsibility of the authors, so questions or comments should be addressed to the corresponding author.

Supplementary_Material
