# Supplementary material for: Bergeyella cardium: Clinical Characteristics and Draft Genome of an Emerging Pathogen in Native and Prosthetic Valve Endocarditis
Source: Open Forum Infect Dis. 2019 Mar 15;6(4):ofz134. doi: 10.1093/ofid/ofz134 (PMC6475584; doi:10.1093/ofid/ofz134)
Supplement: Supplementary_Material [file ofz134_suppl_supplementary_material.doc]

***Bergeyella cardium*: Clinical Characteristics and Draft Genome of an Emerging Pathogen in Native and Prosthetic Valve Endocarditis**

**Supplemental Methods**

Whole Genome Sequencing

100 nanograms of DNA were extracted from the cultured isolate using the Zymo fungal/bacterial DNA kit (Zymo Research) according to the manufacturer’s protocol. Short-read sequencing library was prepared using the NEBNext Ultra II DNA Sequencing kit (New England Biolabs) according to the manufacturer’s protocol and incorporating custom dual unique indexing primers. Paired-end 150 basepair sequencing was performed using an Illumina NextSeq 550 instrument generating 6.3 x 10^6^ raw reads, which were quality filtered using Priceseqfilter (1). Long-read sequencing library was generated using the Rapid Low Input by PCR Barcoding Kit from Oxford Nanopore. This library was sequenced on a MinION instrument and data was basecalled and adaptor-trimmed with Porechop v0.2.3 (2) using default parameters. Hybrid assembly of the short and long reads was performed with Unicycler v0.4.6 using default parameters (3). Assembly yielded 3 contiguous sequences (contigs) 1.83 x 10^6^, 1.21 x 10^5^, and 5.41 x 10^3^ base pairs in length with an average coverage of 920X (Illumina) and 490X (Nanopore) and 1831 predicted coding DNA sequences (CDS) present. Genome annotation was performed using RastK via PATRIC (4). Phylogenetic analysis based on proteins families that are shared across all the genomes was performed using PATRIC (4) with a previously described pipeline (5) consisting of BLAST (6), MCL (7), Muscle (8), hmmbuild (9), hmmsearch (10), Gblocks (11), FastTree (12), and RAxML(13). Percentage identity compared to the draft assembly of *Bergeyella zoohelcum* was determined by aligning the draft assemblies using Mauve v2.4.0 (14) with default settings, and then calculating the percentage of identical bases among the 1.28 megabase assembled regions that overlapped.

**Supplemental References**

1. Ruby JG, Bellare P, Derisi JL. PRICE: software for the targeted assembly of components of Metagenomic sequence data. G3 (Bethesda). 2013 May 20;3(5):865-80.
2. Wick, RR. Porechop. https://github.com/rrwick/Porechop. [Accessed 4 September 2018].
3. Wick RR, Judd LM, Gorrie CL, Holt KE. Unicycler: Resolving bacterial genome assemblies from short and long sequencing reads. PLoS Comput Biol. 2017 Jun 8;13(6):e1005595.
4. Brettin T, Davis J, Disz T, et al. RASTtk: A modular and extensible implementation of the RAST algorithm for building custom annotation pipelines and annotating batches of genomes. Sci Rep. 2015;5:8365.
5. Driscoll T, Gillespie JJ, Nordberg EK, Azad AF, Sobral BW. Bacterial DNA sifted from the *Trichoplax adhaerens* (Animalia: Placozoa) genome project reveals a putative rickettisal endosymbiont. Genome Biol Evol. 2013;5(4):621-45.
6. Boratyn GM, Camacho C, Cooper PS, et al. BLAST: a more efficient report with usability improvements. Nucleic Acids Res. 2013;41:W29-33.
7. van Dongen SM. Graph Clustering by Flow Simulation [dissertation]. Utrecht, Netherlands: University of Utrecht, 2001.
8. Edgar RC. MUSCLE: multiple sequence alignment with high accuracy and high throughput. Nucleic Acids Res. 2004;32:1792-1797.
9. Eddy SR. Profile hidden Markov models. Bioinformatics. 1998;14(9):755-63.
10. Talavera G, Castresana J. Improvement of phylogenies after removing divergent and ambiguously aligned blocks from protein sequence alignments. Syst Biol. 2007;56:564-577.
11. Price MN, Dehal PS, Arkin AP. FastTree 2–approximately maximum-likelihood trees for large alignments. PLoS One. 2010;5:e9490.
12. Stamatakis A. RAxML version 8: a tool for phylogenetic analysis and post-analysis of large phylogenies. Bioinformatics. 2014;30:1312-1313.
13. Rambaut A. FigTree. 2009.
14. Darling AE, Mau B, Perna NT. progressiveMauve: multiple genome alignment with gene gain, loss, and rearrangement. PLoS One. 2010 June 25;5(6):e11147.
